# Adolescent girls and young women: key populations for HIV epidemic control

**DOI:** 10.7448/IAS.18.2.19408

**Published:** 2015-02-26

**Authors:** Rachael C Dellar, Sarah Dlamini, Quarraisha Abdool Karim

**Affiliations:** 1 Centre for the AIDS Programme of Research in South Africa (CAPRISA), Nelson R Mandela School of Medicine, University of KwaZulu-Natal, Durban, South Africa; 2 Department of Epidemiology, Mailman School of Public Health, Columbia University, New York, NY, USA

**Keywords:** HIV prevention, adolescent girls, young women, prevention interventions

## Abstract

**Introduction:**

At the epicentre of the HIV epidemic in southern Africa, adolescent girls and young women aged 15–24 contribute a disproportionate ~30% of all new infections and seroconvert 5–7 years earlier than their male peers. This age–sex disparity in HIV acquisition continues to sustain unprecedentedly high incidence rates, and preventing HIV infection in this age group is a pre-requisite for achieving an AIDS-free generation and attaining epidemic control.

**Discussion:**

Adolescent girls and young women in southern Africa are uniquely vulnerable to HIV and have up to eight times more infection than their male peers. While the cause of this vulnerability has not been fully elucidated, it is compounded by structural, social and biological factors. These factors include but are not limited to: engagement in age-disparate and/or transactional relationships, few years of schooling, experience of food insecurity, experience of gender-based violence, increased genital inflammation, and amplification of effects of transmission co-factors. Despite the large and immediate HIV prevention need of adolescent girls and young women, there is a dearth of evidence-based interventions to reduce their risk. The exclusion of adolescents in biomedical research is a huge barrier. School and community-based education programmes are commonplace in many settings, yet few have been evaluated and none have demonstrated efficacy in preventing HIV infection. Promising data are emerging on prophylactic use of anti-retrovirals and conditional cash transfers for HIV prevention in these populations.

**Conclusions:**

There is an urgent need to meet the HIV prevention needs of adolescent girls and young women, particularly those who are unable to negotiate monogamy, condom use and/or male circumcision. Concerted efforts to expand the prevention options available to these young women in terms of the development of novel HIV-specific biomedical, structural and behavioural interventions are urgently needed for epidemic control. In the interim, a pragmatic approach of integrating existing HIV prevention efforts into broader sexual reproductive health services is a public health imperative.

## Introduction

Southern Africa is at the epicentre of the global HIV epidemic, bearing almost 40% of the global burden of infection despite being home to less than 2% of the global population [1]. In this endemic setting, the dominant mode of transmission is through heterosexual sex. UNAIDS has described the epidemic as a generalized and hyper-endemic to reflect the continued unprecedentedly high (>10%) population prevalence [1,2]. However, generalizability should not be equated to uniformity, as significant heterogeneity exists in terms of where and in whom HIV infections occur, with certain localities and populations being consistently more vulnerable to infection than others [1,3]. Focusing HIV prevention efforts on such high-incidence locations and populations is likely to enable the greatest gains to be made in altering current epidemiological trajectories toward control of the HIV epidemic [4].

**Figure 1:**
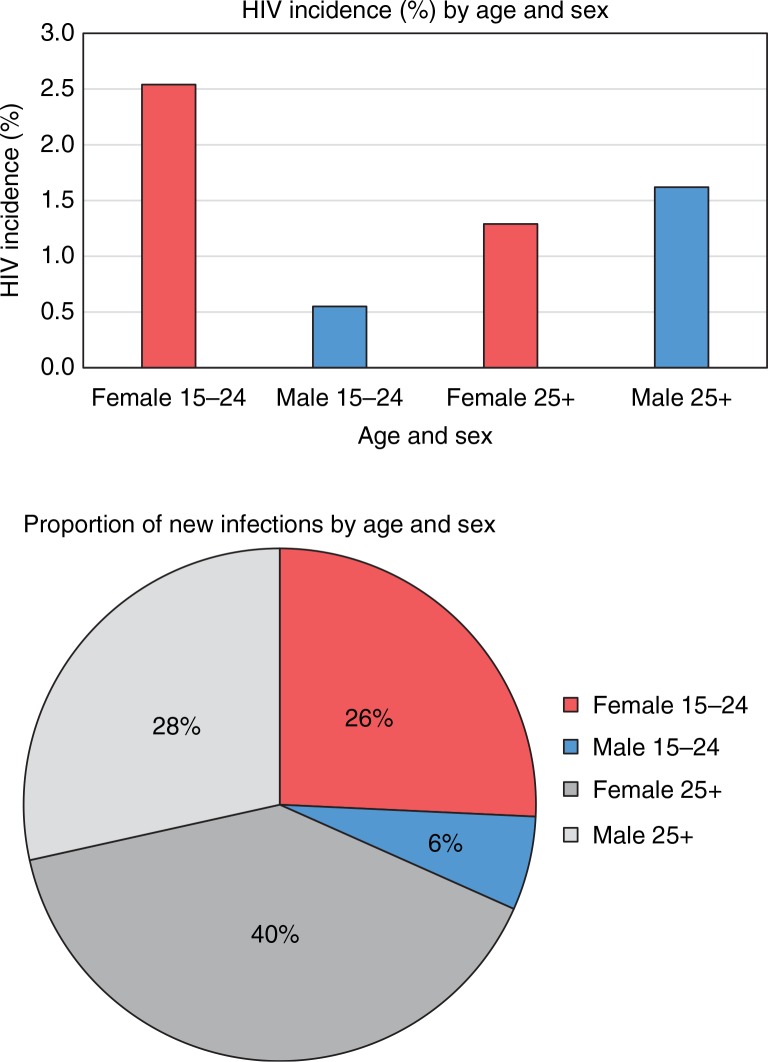
Disproportionate HIV incidence in young women in South Africa. Adapted from Shisana et al. [5].

**Figure 2:**
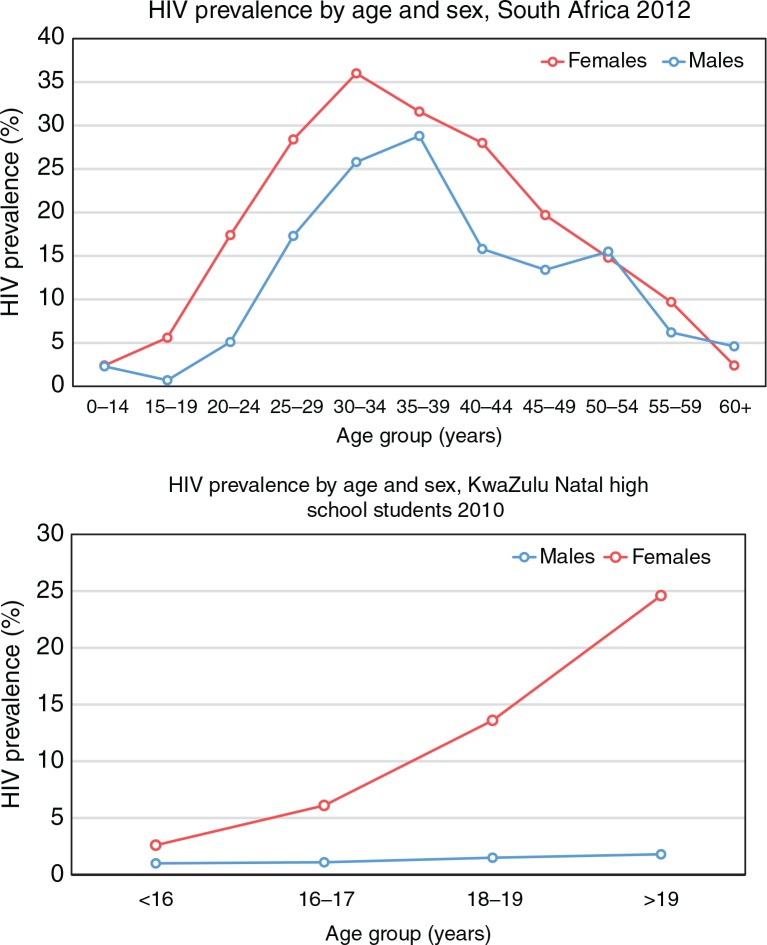
Age–sex disparity in HIV acquisition. Adapted from Shisana et al. [5] and Abdool Karim et al. [8].

An important key population in the southern African setting is young women aged 15–24 years, who contribute nearly 30% of all new HIV infections in the region [1,5,6]. In South Africa, this percentage translates to 113,000 new infections in young women per year, more than four-times the number contributed by their male peers (Figure 1) [5]. Such disproportionately high HIV incidence in young women compared to young men is explained by a striking and characteristic feature of the HIV epidemic in this region: the age–sex disparity in HIV acquisition, wherein young women acquire HIV around five to seven years earlier than young men, often synonymously with sexual debut (Figure 2) [5,7].

As a result of the age–sex disparity in HIV acquisition, HIV prevalence in young women is high, and represents a substantial treatment burden [5,8]; for example, between 2009 and 2013, 27% of women less than 20 years attending antenatal clinics in a rural sub-district of KwaZulu-Natal were found to be HIV positive (unpublished). On a population level, the high incidence in young women is sustaining intergenerational transmission of HIV and contributes to the overall disproportionate burden of HIV in women compared to men [9]. Indeed, approximately 60% of all people living with HIV in sub-Saharan Africa are women [1]. Clearly, achieving the goal of an “AIDS-free generation” depends on reducing the burden of new infection in this key population [10].

However, despite the imperative to prevent HIV acquisition in young women, there remains a paucity of evidence-based interventions available to this population. Indeed, current options are typically limited to promotion of abstinence (or delayed sexual debut), behaviour change, and condom use, all of which are somewhat challenging given the underlying gender-power dynamics of the southern African setting [10]. Further, whilst there has been great optimism following the recent demonstrations of the prevention potential of antiretrovirals (ARVs) – both prophylactically to prevent HIV acquisition (pre-exposure prophylaxis, or PrEP) and for treatment to minimise onward transmission (treatment as prevention, TasP) – to date none of the PrEP trials have included participants <18 years of age, and as such it seems unlikely that these advances will be of benefit to the full range of those considered young women (see Box 1) in the immediate future [10,11].

*Box 1.* Defining young women.

The standard definition of young women includes all those falling within the ages of 15–24 years. As such, most epidemiological data, and much of the discussion here, is presented in terms of this age stratification.

 It is, however, important to note that between these ages, young women undergo significant transitions in lifestyle, maturity, and legal rights which will place them at different vulnerabilities at different time points.

 It is likely that the significance of the <18 years vs. >18 years divide will increase in significance with the rollout of PrEP, as few safety studies for PrEP interventions have been conducted in adolescents <18 years. As such, we would like to encourage the use of this and other sub-strata by those reporting on HIV surveillance in young people.

Moreover, a crucial step in addressing the public health imperative to reduce HIV acquisition in young women is the validation of the safety of existing technologies and interventions for HIV prevention in young women <18 years [10,12]. Concurrently, a concerted effort is required to better understand both the biological and structural factors driving the heightened vulnerability to HIV infection in young women more broadly. Such efforts, in parallel with a consolidation of the evidence obtained from adolescent- and youth-focused HIV prevention interventions and programmes conducted to date, should serve to inform the development of more efficacious interventions.

The objective of this review is to provide an overview of the state-of-the-science of HIV prevention in young women and adolescent girls to inform policy and research direction. Specifically, we aim to (1) summarise the various behavioural and biological factors that predispose adolescent girls and young women to HIV infection, (2) briefly review the evidence from previous HIV prevention interventions targeted toward adolescent girls and young women, and (3) discuss future directions for HIV prevention in adolescent girls and young women.

## Discussion

### Why are adolescent girls and young women so vulnerable to HIV infection?

#### Socio-behavioural associations of HIV infection in adolescent girls and young women

Arguably the most convincing driver of the age–sex disparity in HIV acquisition observed in sub-Saharan Africa is the high prevalence of intergenerational relationships between young women and older men [13,14]. The aggregating prevalence of HIV with increasing age means that, *ceterius paribus*, a young girl engaging in a sexual relationship with an older man is at much higher risk of HIV acquisition compared to a young girl engaging with a male peer (Figure 2) [5]. Further, a young woman engaging in a relationship with an older man may be less likely to negotiate condom use given the gender-power dynamics in the southern African setting, further augmenting her risk [13,15]. Consistent with these data, a number of studies have demonstrated that engagement in an age-disparate or intergenerational relationship is strongly associated with increased HIV prevalence in young women [13,16–18]. Further work is needed to understand how this association may be changing over time with increasing ARV therapy (ART) coverage, and survival of both HIV infected men and women over 25 years of age.

Understanding the complex factors that drive adolescent girls and young women to engage in sexual relationships with older men is challenging, but may be critical in terms of adequately addressing the prevention needs of these key populations. In many cases, young women have reported feeling flattered by the attention of older men, and many relationships are likely to be built on genuine romantic connections [19,20]. In other instances, young women may be motivated primarily by the increased financial or social capital available through engaging in relationships with older men; indeed, many adolescent girls and young women report involvement in these “transactional relationships,” which have significant additional implications for HIV risk [21,22].

Beyond engagement in age-disparate relationships, other risk factors for HIV infection in young women include early sexual debut, few years of schooling, food insecurity, loss of a family member, and experience of gender-based violence [8,17,23–28]. Many of these factors may mediate their effects on HIV acquisition via increasing the relative value of financial capital available through engagement in transactional relationships with older men [21,29–32]. However, independent pathways of risk mediation are also likely to exist. Food insecurity, for example, may also make young women biologically more susceptible to HIV [33].

#### Possible biological mechanisms for heightened vulnerability to HIV infection in adolescent girls and young women

The per-coital act HIV incidence rate in adolescent girls and young women is so high that it seems unlikely that it can be explained by behavioural risk alone [34,35]. Indeed, many young women become infected after just a few coital encounters, and on a population level, acquisition seems almost synonymous with sexual debut [17,36]. As such, there has been significant investigation into potential biological factors that might augment behavioural risk, and a number of mechanisms have been hypothesised to result in heightened vulnerability to infection in young women, compared both to men and to older women.

For example, a number of studies focused on sero-discordant couples have highlighted a higher per-act risk of HIV acquisition in women compared to men [37–40]. A portion of this effect may be attributed to the higher viral load typically observed in men, but the phenomena may also be explained at least in part by physical factors that result in increased exposure to HIV in women, compounded both from the comparatively larger surface area of the cervico-vaginal mucosa and from the increased HIV mucosal exposure time (semen can remain in the female genital tract up to three days post-coitus) [41,42]. The higher per-act risk of HIV acquisition in women could also result from the relatively high levels of activation of the immune cells in the female genital tract, the increased expression of HIV co-receptors in cervical cells compared to foreskin cells, and/or a mucosal surface more likely to acquire micro-abrasions during sex: together, these factors result in more accessible portals for HIV entry in women [35,43–46].

Further, young women are more susceptible to HIV infection compared to older women, and there are a number of biological factors that have been promulgated to explain this age-variability in vulnerability. For example, the immature cervix has a greater proportion of genital mucosa exposed to HIV that is highly susceptible to infection, and young women have relatively high levels of genital inflammation which have consistently been reported to increase HIV acquisition risk [23,35,47–49].

**Figure 3:**
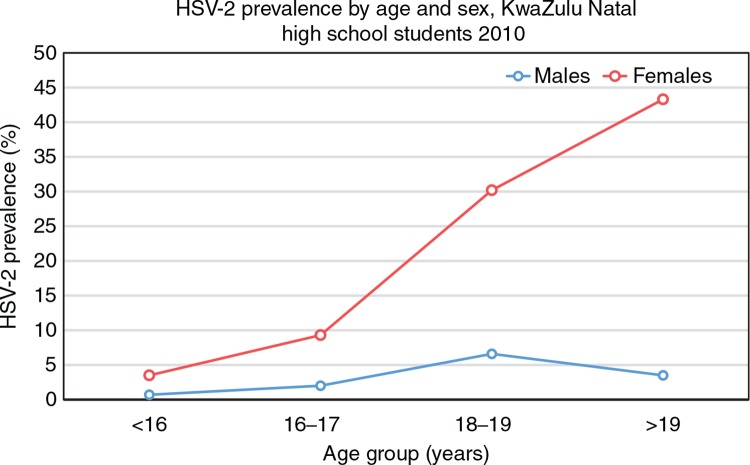
Age–sex disparity in HSV-2 acquisition. Adapted from Abdool Karim et al. [8].

When considering the apparently uniquely high per-act HIV acquisition risk in young women, it is also necessary to consider other relevant contextual factors that may mediate the infection environment, including other sexually transmitted infections (STIs) and contraceptive use. For example, many bacterial and viral STIs are associated with increased risk of HIV infection, and are much more prevalent in young women compared to young men [50,51]. A recent school-based survey conducted in rural KwaZulu-Natal, South Africa, found the trend in herpes simplex virus-2 (HSV-2) acquisition to mirror the age–sex disparity in HIV infection, with young female students acquiring HSV-2 soon after sexual debut, and a more than three-fold higher prevalence of HSV-2 compared to their male peers (Figure 3) [8]. Interestingly, recent HSV-2 infection may confer the greatest impact in terms of increasing vulnerability to HIV, such that the female genital tract in the immediate years following HSV-2 acquisition may be particularly susceptible to HIV infection [52,53].

Beyond STIs, other biological risk factors may also be amplified in young women. For example, one study has shown that the use of the hormonal contraceptive depot medroxyprogesterone acetate (DMPA) increases the risk of HIV acquisition in young women (18–24 years), while decreasing HIV acquisition risk in older women (≥25 years) [54]. Further, although establishing causal relationships is challenging, intra-vaginal cleaning practices are more prevalent in younger women, suggesting these women are consequently more likely to have an altered vaginal flora, potentially heightening their HIV susceptibility [55,56].

Together these biological factors may create a “perfect storm” of conditions in recently sexually debuted adolescent girls and young women in southern Africa making them uniquely vulnerable to HIV infection when exposed to the virus via engaging in unprotected sex with an HIV-positive partner.

### Effectiveness of current HIV prevention interventions available to adolescent girls and young women

#### In-school interventions

Schools provide convenient venues for HIV prevention education, and not surprisingly a vast number of youth-targeted HIV, STI, and pregnancy prevention programmes operate in schools throughout sub-Saharan Africa [57]. The effectiveness of such programmes in young people in sub-Saharan Africa has been the subject of a considerable number of systematic reviews [58–71]. To summarise the evidence, several programmes have been demonstrated to be effective in improving knowledge and attitudes concerning HIV and the uptake of HIV testing. These data follow a general trend in sub-Saharan Africa of increasing comprehension and understanding about HIV in young people [1]. Those interventions demonstrating the most success are characterised by a number of factors, including but not limited to: iterative and context-specific session programmes, HIV prevention and sexual and reproductive health (SRH) curricula that include tasks focused toward more general skills and knowledge development, and delivery by trained facilitators [57]. In contrast, abstinence-only and peer-led in-school interventions tend to be ineffective [57,62].

Despite some apparent successes, few rigorously conducted trials have assessed the impact of interventions on biological outcomes, including HIV, STI and/or pregnancy incidence. Those trials that have demonstrated no significant effects of any school-based intervention on these biological outcomes, in spite of reporting positive impacts on self-reported behaviour change in adolescents [72–74]. These results may stem from the relatively strong prevention effect of being in school itself, which may dwarf the effect of any behavioural intervention. However, the burden of HIV in school-attending adolescents, while lower than out-of-school adolescents, remains significant, and thus there is also concern that the results might point to differential desirability bias by trial arm, which questions the validity of significant changes in self-reported markers of behaviour change reported by other studies. The data from school-based trials also underscore that while knowledge is a pre-requisite for HIV prevention, it is in itself insufficient to prevent HIV infection.

#### Attempts to make health services youth-friendly

Other interventions to prevent HIV infection in young people have focused on health systems strengthening in an effort to address barriers to healthcare access by increasing the provision of high-quality, youth-friendly HIV and SRH services. Such interventions are potentially critical, as there is significant demand for more comprehensive SRH services that recognise the inter-relationships between HIV and broader SRH and thus the importance of integrated service delivery [1,57].

Interventions to make health services more youth-friendly have typically focused on a different combinations of training of service providers, outreach activities, and provision of mobile services targeted toward specific high-risk adolescent populations [66,75–77]. Many of these interventions have been successful in terms of increasing uptake of services by young people. However, similarly to in-school interventions, there is a notable dearth of biological-outcome-based assessment.

#### Community-level interventions

HIV prevention interventions implemented at the community level are highly heterogeneous, including sporting events, mentoring and youth centres [78]. Evaluation of these interventions highlights their largely positive impact on knowledge and attitudes to HIV. However, these interventions often fail to reach the most HIV vulnerable populations, and evaluation designs are generally weak. Only one study to our knowledge has assessed HIV incidence, and this study reported no evidence of effectiveness [79].

#### Conditional cash transfers

Cash transfers to young people that incentivise safer behaviour have recently emerged as a new strategy to reduce young people's vulnerability to HIV [1,80]. The evidence in support of the efficacy of this strategy is limited but promising. Indeed, a recent randomized controlled trial in Lesotho demonstrated that a programme of financial incentives reduced the probability of acquiring HIV by 25% over two years [81]. Similarly, an independent randomized controlled trial in Malawi reported that those female high school students who received conditional cash transfers (CCTs) were 64% less likely to be HIV infected compared to those who were not [82]; however, baseline HIV infection was not measured. These data suggest a potential for CCT to prevent HIV in young people, and outcomes of current research in the field such as HPTN 068 are eagerly awaited.

### Gaps and future directions

Despite the large and immediate need for HIV prevention in adolescent girls and young women, there is a dearth of evidence-based interventions available to them to reduce their risk. Given the diversity of epidemics within and between countries, in order to develop more efficacious youth-focused prevention interventions, a sound understanding of the local epidemic is required as well as the bio-behavioural nexus that renders adolescent girls and young women more vulnerable to HIV infection. The significant SRH needs of young women should be central to the design of new interventions, as integration of services is the backbone of a pragmatic approach to address needs now, even as we refine, develop and test new and novel approaches [1,83]. A careful review of previous interventions and their evaluations is needed to ensure maximum gains. Most notably, it is critical that any future intervention should be rigorously assessed for effectiveness in controlled trials with biological outcomes prior to wide-scale implementation to maximise efficiency and effectiveness of resource allocation. Many researchers would benefit from engaging the young women themselves as partners in intervention design and implementation, and certainly encouraging male partner buy-in and female empowerment will also be important in those settings where gender-power dynamics augment HIV risk.

A further important direction for future research should be to develop interventions targeted to hard-to-reach young people who might be missed by school- or community-based interventions. The evidence for the best practice in reaching such populations is particularly limited, despite their often greater risk of HIV acquisition. However, our own experiences highlight that some important components of making service provision palatable and attractive to hard-to-reach adolescents include anonymized testing, flexible clinic hours and adaptions of respondent-driven sampling. Concurrently, efforts should be made to keep adolescents in school. The task of developing and evaluating new HIV prevention interventions – particularly those programmes that aim to address the underlying social vulnerabilities – is substantial, and will potentially require decades of concentrated action, during which time adolescent girls and young women will continue to become infected in their hundreds of thousands. As such, it is a moral imperative to effectively deliver what we know works now. The most pressing example of a technology that we know works but is not being delivered is PrEP, which was developed specifically with young women in southern Africa in mind: designed to allow them to exercise their rights over their health and take control over their own risk without dependence on their sexual partners. While the number of randomized controlled trials demonstrating the effectiveness of PrEP continues to grow, this success has yet to be translated into product availability in southern Africa. Undeniably, PrEP is not 100% effective, is limited by adherence and would benefit from improvements currently in development; however, one has to question where the threshold of evidence required for rollout of current forms of PrEP to young women in southern Africa lies. A simple calculation highlights that even with a 39% efficiency, rollout of Tenofovir gel to young women aged 15–24 years in South Africa alone might prevent more than 44,000 infections in one year. Implementation and policy science are urgently needed to translate research on PrEP effectiveness into averted infections. Further, there is also work to be done in ensuring that on rollout, the state-of-the-science of prevention is not lagging behind in adolescents <18 years because of restrictive ethico-legal guidelines that often prevent them from participating in biomedical research in spite of their substantial need [6,10].

This review was restricted to considering HIV prevention in adolescent girls and young women. However, the treatment needs resulting from the unprecedentedly high HIV incidence rates in these key populations should not be underestimated: in Lesotho for example, almost a quarter of all young people aged 15–24 years are infected with HIV [1]. Adolescent-focused HIV prevention interventions should also seek to meet the needs of HIV-positive young people who face significant barriers to care. Indeed, of note is that adolescents (10–19 years) are the only age group in which AIDS deaths have risen between 2001 and 2012 [1].

## Conclusions

Meeting the HIV prevention and SRH needs of adolescent girls and young women who are at uniquely high risk of HIV acquisition is a public health and moral imperative and a requirement to meet the laudable goals of achieving an AIDS-free generation and/or epidemic control. However, despite this imperative, evidence-based prevention options available to adolescent girls and young women remain limited, and even as efforts get underway to develop more efficacious interventions, they are likely to take many years to reach fruition. Immediate action is therefore needed to facilitate this key population to mediate their own risk, including as first steps rollout of PrEP, adolescent enrolment in biomedical HIV prevention trials, and provision of accessible and integrated SRH-HIV prevention services.

## Author information

RD is a research fellow at CAPRISA supported by the CAPRISA Training Programme.
